# Cycling GLP-1 receptor agonist treatment induces therapeutic resistance and increased adiposity

**DOI:** 10.1172/jci.insight.205174

**Published:** 2026-03-31

**Authors:** Anna J. Son, Emmanuel Rapp, Alex Wiezorek, Max G. Leung, Ronadip R. Banerjee, Thomas H. Leung

**Affiliations:** 1 Department of Dermatology, University of Pennsylvania, Philadelphia, Pennsylvania, USA.; 2 Division of Endocrinology, Department of Medicine, University of Arizona College of Medicine, Tucson, Arizona, USA.; 3 Corporal Michael J. Crescenz Veterans Affairs Medical Center, Philadelphia, Pennsylvania, USA.

**Keywords:** Endocrinology, Metabolism, Clinical practice, Obesity

## Abstract

Stopping and restarting GLP-1 weight-loss drugs in mice made them less effective over time and increased fat gain compared with continuous treatment.

**To the Editor:** GLP-1 receptor agonists (GLP-1 RAs) have revolutionized obesity management, yet “real-world” use is characterized by frequent treatment discontinuation and reinitiation (1, 2). More than half of patients discontinue therapy within 24 months, and many subsequently restart (1). Because weight regain is rapid following cessation, and up to 40% of GLP-1 RA–induced weight loss reflects lean mass, repeated cycling may exert a cumulative metabolic toll given the limited capacity for skeletal muscle recovery (3–5). Here, we define the metabolic impact of GLP-1 RA cycling in vivo.

We modeled intermittent GLP-1 RA therapy in diet-induced obese male C57BL/6J mice. Mice assigned to continuous treatment received daily semaglutide for 4 months. In contrast, the cycled group underwent 2 rounds of 2-week semaglutide exposure followed by 2-week withdrawal before transitioning to continuous treatment (Figure 1A and Supplemental Methods; supplemental material available online with this article; https://doi.org/10.1172/jci.insight.205174DS1).

During the initial treatment phase (cycle 1), weight loss was comparable between groups. Upon semaglutide withdrawal, cycled mice rapidly regained weight. Reinitiation of semaglutide (cycle 2) induced weight loss; however, cycled mice failed to reach the prior weight nadir achieved during cycle 1 (Figure 1B). After a second withdrawal period, body weight in cycled mice rebounded to 7% above baseline. A third course of semaglutide (cycle 3) also produced a markedly attenuated weight loss response, and cycled mice weighed 20% more than continuously treated controls (Figure 1, B and C, and Supplemental Figure 1, A and B). Notably, this “therapeutic resistance” persisted despite 62 days of uninterrupted semaglutide treatment (days 58–120).

Body composition analysis by EchoMRI (day 83) demonstrated increased total and percentage fat mass and reduced percentage lean mass in cycled mice versus continuously treated controls, while absolute lean mass was unchanged (Figure 1D and Supplemental Figure 1C). At study end (day 120), cycled mice exhibited expansion of epididymal white adipose tissue (eWAT), inguinal white adipose tissue (iWAT), and brown adipose tissue (BAT) depots, accompanied on histology by adipocyte hypertrophy (Figure 1, E and F, and Supplemental Figure 1, D and E). Skeletal muscle weights were comparable between groups (Supplemental Figure 1F). Together, these findings indicate that intermittent semaglutide exposure preferentially promotes adipose accrual despite preservation of absolute lean mass.

Metabolic phenotyping revealed increased serum leptin and impaired glucose tolerance in cycled mice (Figure 1G and Supplemental Figure 1G). In contrast, insulin tolerance testing revealed no difference between groups, suggesting that the observed glucose intolerance is not driven by peripheral insulin resistance (Supplemental Figure 1H). Indirect calorimetry revealed no differences in oxygen consumption (VO_2_), carbon dioxide production (VCO_2_), energy expenditure, food or water intake, or locomotor activity between groups. However, respiratory exchange ratio (RER) was reduced in cycled mice, consistent with a shift toward increased lipid utilization (Supplemental Figure 2). Increased lipid utilization in the absence of weight reduction suggests a compensatory metabolic adaptation rather than enhanced energy expenditure. Because differences in body composition are intrinsic to the cycling phenotype, these studies assess the integrated metabolic consequences of treatment interruption but do not distinguish primary effects on energy balance from secondary effects of altered lean and fat mass.

A second independent cohort reproduced these findings (Figure 1, H and I, and Supplemental Figure 3). Following semaglutide withdrawal (cycle 2), cycled mice again exhibited rapid weight regain, reaching 9% above baseline. Reinitiation of treatment (cycle 3) resulted in attenuated weight loss, with cycled mice remaining 20%–25% heavier than continuously treated controls (Figure 1J).

Taken together, these data demonstrate that intermittent GLP-1 RA exposure rapidly attenuates therapeutic efficacy, with loss of response evident by the second cycle and resulting in approximately 20% higher body weight after 3 exposures compared with continuous treatment. We propose that repeated treatment interruptions lead to a cumulative deficit in lean mass that is not fully recovered during off-treatment intervals. Consequently, each successive cycle begins from a progressively altered body composition characterized by increased adiposity relative to lean mass. Under these conditions, homeostatic mechanisms may preferentially defend remaining lean mass, establishing a physiological constraint that limits further weight loss despite continued GLP-1 RA exposure. Future studies are needed to define these molecular mechanisms. Jiang and colleagues recently reported similar attenuation of weight loss using liraglutide in aged UM-HET3 mice (6). Despite differences in mouse strain, age, and GLP-1 RA used, the concordance between studies suggests that this attenuation of efficacy may represent a class effect rather than a drug-specific phenomenon. As both studies were performed in obese male mice, validation in females and in human populations will be essential to determine translational relevance. These findings raise concern that cycling GLP-1 RA may promote therapeutic resistance and unfavorable metabolic outcomes, with important implications for long-term clinical management and treatment durability.

## Conflict of interest

The authors have declared that no conflict of interest exists.

## Funding support

- This work is the result of NIH funding in part and is subject to the NIH Public Access Policy. Through acceptance of this federal funding, the NIH has been given a right to make the work publicly available in PubMed Central.
- The Berstein Family (to THL).
- NIH grant R01AR079483 (to THL).
- US Department of Veterans Affairs grant I01RX002701 (to THL).
- Edwin and Fanny Gray Hall Center for Human Appearance (to THL).
- Montague Fellowship (to THL).
- H.T. Leung Foundation (to THL).
- NIH grants R01DK120761 and R21HD111996 (to RRB).

## Supplementary Material

Supplemental data

Supporting data values

## Figures and Tables

**Figure 1:**
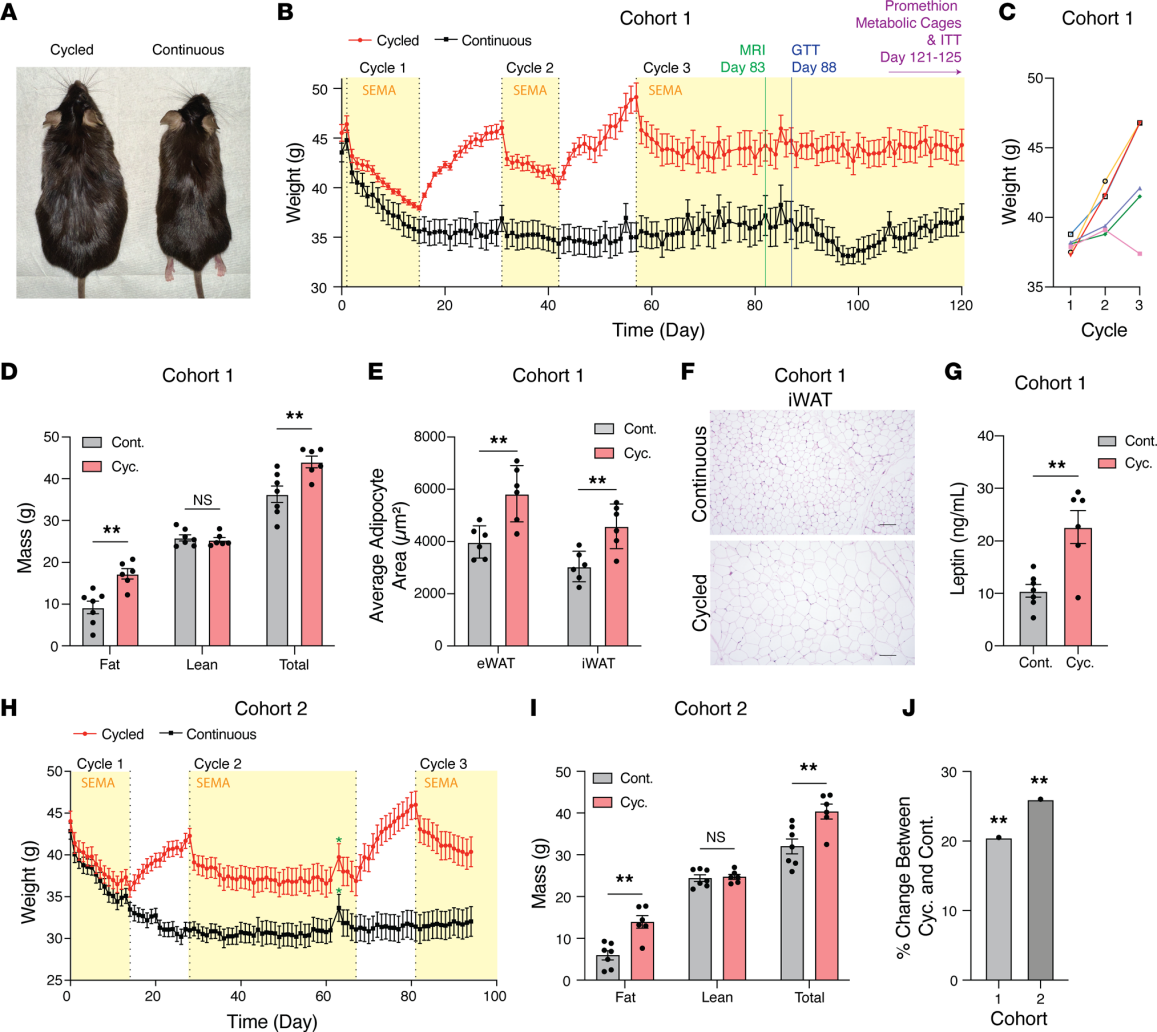
Cycling GLP-1 RA treatment induces therapeutic resistance and rebound obesity. (**A**) Representative image of cycled and continuous semaglutide-treated (SEMA-treated) mice on day 120. (**B**) Body weight trajectories of Cohort 1 cycled (red, *n* = 6) and continuous (black, *n* = 7) mice. GTT, glucose tolerance testing; ITT, insulin tolerance testing. (**C**) Body weights of individual cycled mice 14 days after each cycle. (**D**) Fat, lean, and total mass by EchoMRI on day 83. (**E**) Mean adipocyte area in eWAT and iWAT, quantified from representative H&E-stained sections (**F**) (scale bar, 100 μm). (**G**) Plasma leptin levels in Cohort 1. (**H**) Body weight trajectories of Cohort 2 cycled (red, *n* = 6) and continuous (black, *n* = 7) mice over 95 days; green star, 1 missed treatment day. (**I**) Fat, lean, and total mass by EchoMRI on day 95. (**J**) Percentage weight change in cycled versus continuous mice across both cohorts. Unpaired 2-tailed Student’s *t* test. NS, not significant. *P < 0.05, **P < 0.01. Data presented as mean ± SEM.

## Version 1 03/31/2026

In-Press Preview

## Version 2 05/22/2026

Electronic publication
