# Supplementary material for: Cycling GLP-1 receptor agonist treatment induces therapeutic resistance and increased adiposity
Source: JCI Insight. 2026 Mar 31;11(10):e205174. doi: 10.1172/jci.insight.205174 (PMC13232713; doi:10.1172/jci.insight.205174)
Supplement: Supplemental data [file jciinsight-11-205174-s056.pdf]

**Supplemental Data**

**Methods**

**Sex as a biological variable**

*This study examined male mice. We do not anticipate any differences between male and female mice.*

**Mouse Models**

*Male, diet-induced obese mice on a C57Bl/6J background (Jackson Laboratory, #380050, 16-weeks) were maintained on 60% high-fat diet (HFD, Research Diets, D12492) beginning at 6-weeks of age. Semaglutide (Peptide Sciences) was administered at 120ug/kg by daily subcutaneous injection. Mice were euthanized in a fed state, and tissue and plasma were collected for further analysis.*

**Metabolic rate and activity measurement**

*Metabolic rate and activity were measured by in vivo indirect calorimetry using the Prometheon metabolic cage system by the Rodent Metabolic Phenotyping Core. Following a 2-day acclimation period, data was collected from singly-housed mice every fifteen minutes by Oxymax software and analyzed using the CalR 2 application.*

**EchoMRI**

*Whole-body total, fat and lean mass were measured noninvasively using quantitative magnetic resonance (EchoMRI, Echo Medical Systems). Conscious mice were briefly restrained, and measurements were acquired according to the manufacturer's instructions.*

**Glucose (GTT) and Insulin (ITT) Tolerance Testing**

*These assays were performed by the Rodent Metabolic Phenotyping Core. Mice were fasted for 6 hours GTT or 4 hours ITT, with free access to water prior to testing. Baseline blood glucose was measured from tail vein blood using a handheld glucometer. Mice received an intraperitoneal injection of D-glucose (1*

g/kg body weight), or human insulin (0.75 U/kg body weight) for GTT and ITT respectively. Blood glucose concentrations were measured at post injection timepoints.

**Serum leptin**

Serum leptin levels were measured by ELISA (KE10048, Proteintech) per manufacturer's directions.

**Histology and Image Analysis**

eWAT and iWAT depots were fixed overnight in 4% paraformaldehyde and processed by the NIH-funded Skin Biology and Disease Resource Center Core. Tissues were dehydrated through graded ethanol (70%, 85%, 95%, and 100%), embedded in paraffin, sectioned, and stained with hematoxylin and eosin. Images were acquired at 10× magnification using a Keyence BZ-X700 microscope. Adipocyte area was quantified with the Adiposoft plugin in ImageJ using one image per animal, and mean adipocyte size was reported.

**Statistics**

Experiments were not randomized, and investigators were not blinded to allocation during experiments and outcome assessment, unless noted in the text. Food and water intake, energy expenditure, VO□, and VCO□ were analyzed by ANCOVA with lean mass as a covariate. Locomotor activity, energy balance, and RER were analyzed by one-way ANOVA. All indirect calorimetry analyses were performed using CalR 2. GTT and ITT were analyzed by two-way ANOVA with mixed-effects model and group differences at individual time points assessed using Šidák-corrected post hoc tests. All other two-group comparisons were performed using two-tailed unpaired Student's t-tests. In all tests, a p-value of less than 0.05 is considered significant. Higher levels of significance are indicated as follows: ** $p < 0.01$, *** $p < 0.001$, **** $p < 0.0001$ in the text.

**Study Approval**

*Mouse studies were approved by the Institutional Animal Care and Use Committee of the University of Pennsylvania, Philadelphia, PA. (Protocol #805620 issued to Thomas Leung).*

**Data Availability**

*Raw data for all figures can be found in the Supporting Data Values file.*

**Author contributions:**

*Experiment conceptualization and methodology performed by: R.R.B., and T.H.L.*

*Investigation performed by A.S., E.R., A.W., M.L., T.H.L.*

*Writing of the manuscript performed by: T.H.L.*

*Reviewing and editing the manuscript performed by: E.R., T.H.L., R.R.B.*

*First author order was determined by first name alphabetical order with A.S. listed first.*

**Acknowledgments:**

*We thank Mitchell Lazar for reagents and helpful discussions. We thank the Rodent Metabolic Phenotyping Core supported in part by NIH grant S10-OD025098, the Cox Institute, and the Institute for Diabetes, Obesity and Metabolism for performing indirect calorimetry and glucose and insulin tolerance tests. We thank the Penn Skin Biology Disease Resource Center (SBDRC) supported in part by NIH grant P30-AG059300 and Department of Dermatology for performing histology.*

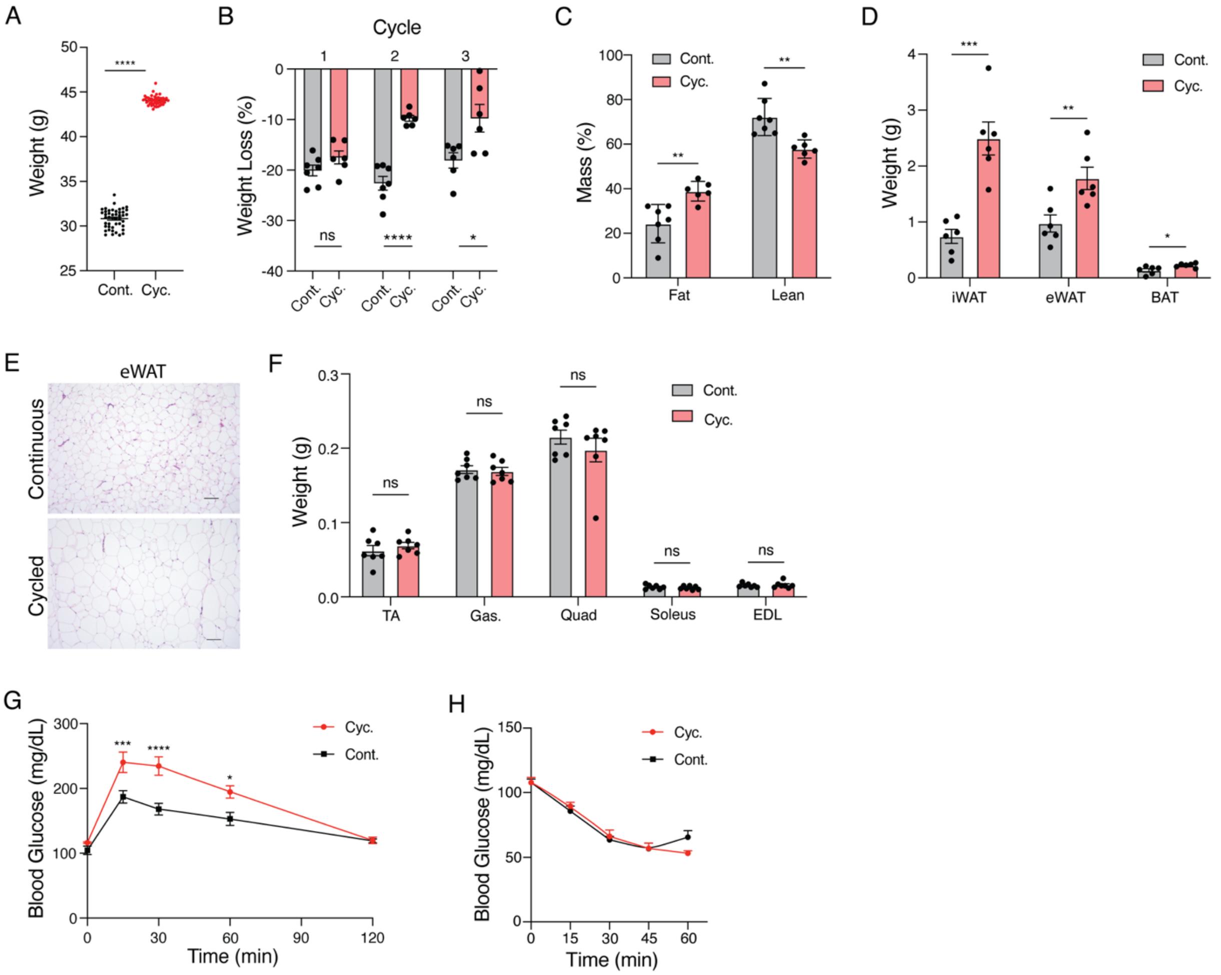


**Supplementary Figure 1. Comparing continuous and cycling semaglutide treatment in cohort 1.** **(A)** *Dot plot comparing average body weights (g) of cycled and continuous groups from day 76 to 120.* *(***B***) Percent weight loss relative to weight at the beginning of each treatment cycle. (***C***) Fat and lean mass percentages measured by EchoMRI at day 83. (***D)** *Weights of dissected iWAT, eWAT, and BAT depots.* *(***E***) Representative H&E-stained sections of eWAT. Scale bars, 100 µm. (***F***) Weights of dissected muscles. TA, tibialis anterior; Gas, gastrocnemius; Quad, quadriceps; EDL, extensor digitalis longus. (***G***) Glucose tolerance test (GTT) curves. (***H***) Insulin tolerance test (ITT) curves. Panels G and H were analyzed by Two-way ANOVA. Panels A, B, C, D, and F were analyzed by unpaired two-tailed Student's t test. ns, not significant, *p <0.05, **p <0.01, ***p<0.001, ****p <0.0001. Mean ± SEM are plotted.*

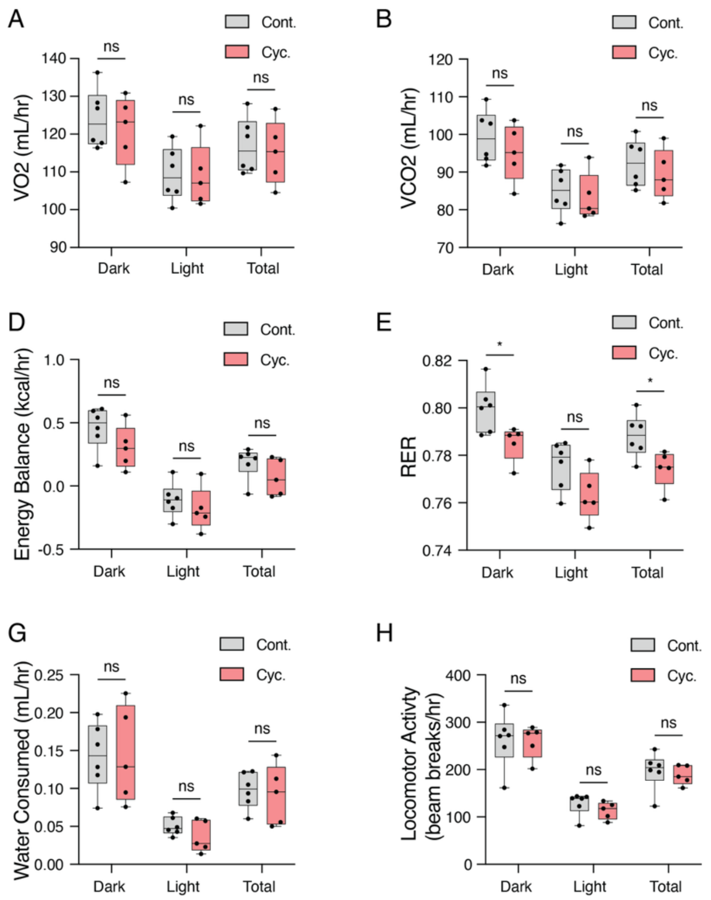


**Supplementary Figure 2. Indirect calorimetry measurements in cohort 1.** *(***A-H***) Box plots comparing cycled (n=5) versus continuous (n=6) during light hours, dark hours, and total day for each parameter are represented. (***A***) Oxygen Consumption (mL/hr). (***B***) Carbon Dioxide Production (mL/hr). (***C***) Energy Expenditure (kcal/hr). (***D***) Energy Balance (kcal/hr). (***E***) Respiratory Exchange Ratio (RER). (***F***) Food Intake (kcal/hr). (***G***) Water Intake (mL/hr). (***H***) Locomotor Activity (beam breaks/hr). Presented data are raw values. Statistical comparisons for Panels A and B were performed by ANCOVA with lean mass as a covariate. Statistical comparison for Panels C-H were analyzed by one-way ANOVA. ns, not significant and *p <0.05. Mean ± SEM are plotted.*

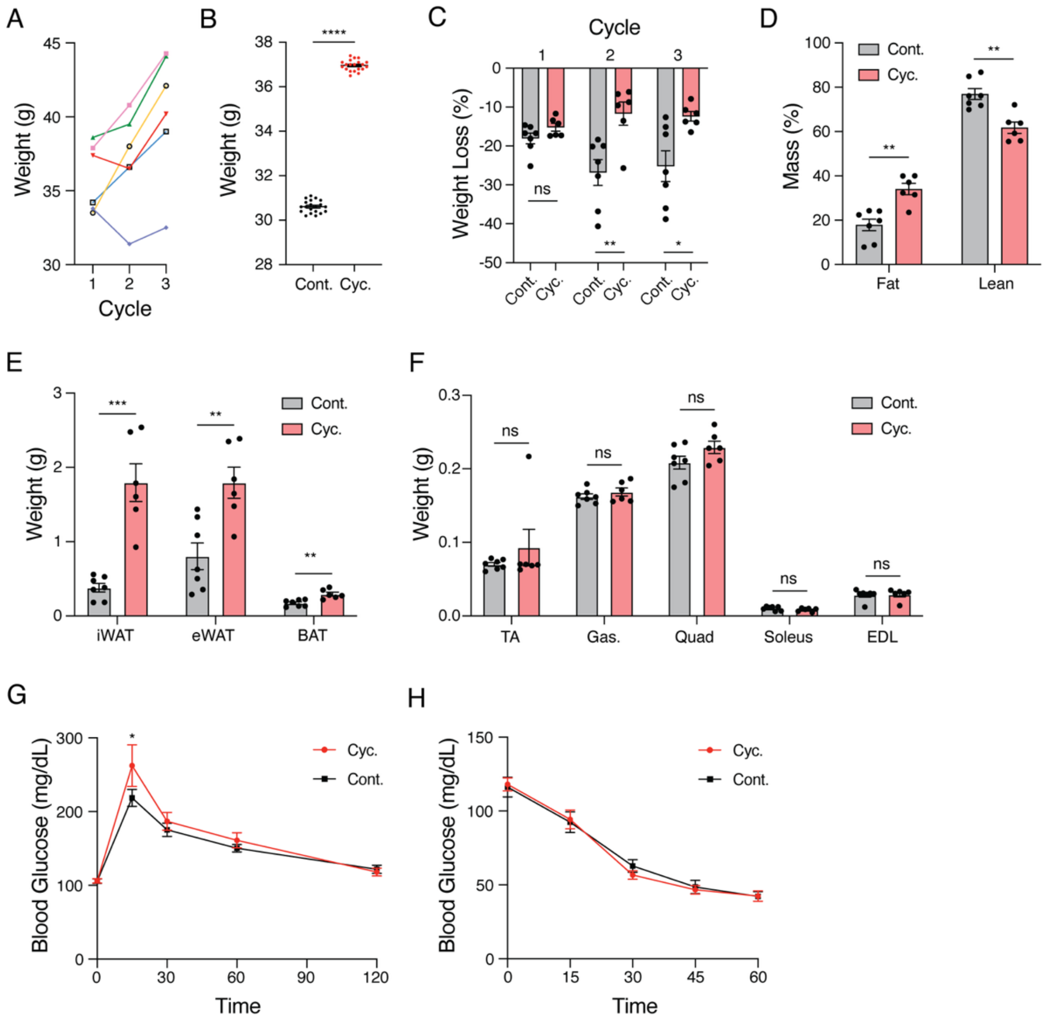


**Supplementary Figure 3. Comparison of continuous and cycling semaglutide treatment in cohort 2.** *(***A***) Body weights of cycled mice 14 days after each cycle.* **(B)** *Dot plot comparing average body weights (g) of cycled and continuous groups from day 42 to day 61.* **(C)** *Percent weight loss relative to weight at the beginning of each treatment cycle. (***D***) Fat and lean mass percentages measured by EchoMRI at day 95. (***E***) Weights of dissected iWAT, eWAT, and BAT depots. (***F***) Weights of dissected muscles. TA, tibialis anterior; Gas, gastrocnemius; Quad, quadriceps; EDL, extensor digitalis longus. (***G***) Glucose tolerance test (GTT) curves. (***H***) Insulin tolerance test (ITT) curves. Panels G and H were analyzed by two-way ANOVA. Panels B-F were analyzed by unpaired two-tailed Student's t test. ns, not significant, *p <0.05, **p <0.01, ***p <0.001, ****p <0.0001. Mean ± SEM are plotted.*
